# Two Remarkable Spiders

**Published:** 1891-05

**Authors:** 


### TWO REMARKABLE SPIDERS.

The *Mygalidæ*, or trap-door spiders, are a widely scattered species, and are particularly remarkable for the wonderful nests which they build instead of spinning a web. The *Cteniza Californica*, popularly known as the tarantula, digs a hole in the ground, about an inch in diameter, and from two inches to a foot in depth. It is lined on the inside with a fine, soft silk, spun by the spider, and the mouth, which is slightly enlarged, is closed by a cover which moves on a silken hinge, and fits so closely to the opening, when closed, that not even a knife blade can be inserted between it and the sides of the tube. This cover is made of dirt fastened together with threads, and, although lined with silk on the inside, is covered on the outside with sand, dirt, moss, etc., so that it exactly resembles the surrounding soil and is almost impossible to discover. In addition, the under side of the cover is provided with small holes, into which the spider inserts her claws, and by which she can hold it down so firmly that it is impossible to raise it without tearing. In these nests the spiders live, only coming out in search of prey. The eggs are laid and hatched in the nest, which serves as a nursery for the young spiders till they are old enough to go and dig for themselves.

The shape and construction of these nests varies greatly with different species. The genus *Atypus* extends the silken lining of the tube several inches outside the ground, where it rests among the stones and plants; other spiders make a second door, half way down the nest; others build the nest with a branch, or chamber, into which they can retreat if an enemy succeeds in forcing the outer entrance. This chamber is sometimes closed by another trap-door, and sometimes communicates with the air by a concealed opening. The *Mygalidæ* are certainly most remarkable animals, and their constructive skill—which exceeds that of many men—is hard to account for. The idea of "instinct" explains nothing, but is simply equivalent to saying that we don't know; and the source of their wonderful knowledge must be left for future investigators to discover.

The *Argyronetes*, or water spiders, are even more extraordinary in their habits than their earth-dwelling cousins. Although they are true air breathing animals like other spiders, for some unaccountable reason they prefer to live under the water among the fishes, where they spin a web in the shape of a bag, with the opening on the under side, just like a diving bell, and, having filled it with air, they lay their eggs and bring up their young in their cosy subaqueous home, finding plenty of food in the insects which live among the aquatic plants. The body of these spiders is covered with fine hairs, which prevent the water from touching the body, and, by their total reflection of light, give them a silvery appearance when in the water—whence their name.

The manner in which this spider fills her nest with air is most curious. After spinning the threads, the spider goes to the surface of the water, and, by a quick movement of the hind legs, catches a small bubble of air between them and the abdomen, with which she descends to the nest, into which it is liberated. After this process has been patiently repeated many times, the nest becomes full of air, and is ready for occupancy.

It must not be supposed that these nests as constructed are perfectly air-tight. The small meshes are quite sufficient to retain the air bubble and prevent it from rising to the surface. This phenomenon is due to the peculiar and complex principle of surface tension, by which the thin film at the surface of a liquid acts, in a certain sense, like a solid covering. By an application of the same principle a cylinder of wire gauze or even a fine sieve, may be filled with water, and needles, or a steel pen, be made to float, if carefully placed upon its surface. Those insects which walk so easily over the surface of ponds and streams are indebted to this same principle for their support, and not to the inherent buoyancy of their bodies.

